# Herpes simplex virus infection: Management of primary oral lesions in children

**DOI:** 10.1002/ccr3.6127

**Published:** 2022-08-03

**Authors:** Chaima Khalifa, Afef Slim, Garma Maroua, Sameh Sioud, Hajer Hentati, Jamil Selmi

**Affiliations:** ^1^ Department of Oral Medicine and Oral Surgery Dental Clinic of Monastir Monastir Tunisia; ^2^ Faculty of Dental Medicine, Laboratory of Oral Health and Maxillofacial Rehabilitation (LR12ES11) University of Monastir Monastir Tunisia

**Keywords:** gingivostomatitis, herpes simplex virus type 1, herpetic withlow, infection, oral

## Abstract

Primary herpetic gingivostomatitis (PHGS) represents the most observed clinical feature of primary herpes infection with the simplex virus (HSV). It is often caused by HSV‐1 and affects children most of the time. Unlike, the majority of primary HSV infections that is asymptomatic. It may be preceded by some prodromal symptoms like fever, anorexia, irritability, malaise, and headache. After the resolution of the primary infection, the virus remains latent in a nervous ganglion. The aim of the present paper was to report a case of severe PHGS with herpetic whitlow in a 10‐year‐old child.

## Graphical abstract

Herpes simplex virus type 1 (HSV‐1) is common in children and gives rise to a variety of clinical disorders. It should not be misdiagnosed or attributed to teething. Parents should be aware of its contagiousness. Meticulously identifying this pathological entity provides an adequate treatment.

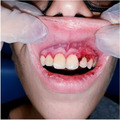

## 1 INTRODUCTION

Herpes is one of the most common chronic viral infections in humans. This virus has the property of being able to remain latent in nerves and ganglion cells; and to cause possible recurrences. There are two main types of herpes virus; HSV‐1 (Herpes simplex virus 1) cause oral infections and HSV‐2 affects the genital tract.^1^ The contamination, strictly human, is due to HSV‐1. It is transmitted through direct contact with a person during the primary infection, recurrence, or asymptomatic viral excretion.^1^ The first contact with HSV‐1 occurs mainly during childhood, especially between 6 months and 3 years after the loss of maternal antibodies.^2^ This infection is most often asymptomatic, or almost, with rough clinical manifestations confused with those of dental eruption. But in 25–30% of cases, the first contact results in gingivostomatitis or painful pharyngitis.^3^ The virus then remains quiescent in a nervous ganglion in the cephalic region. When there is a trigger like sun, cold, infection, stress, or menstruation, recurrence is possible. Herein, we present a case of a herpetic primary infection in a 10‐year‐old child with strong systematic symptoms.

## 2 CASE REPORT

**FIGURE 1:**
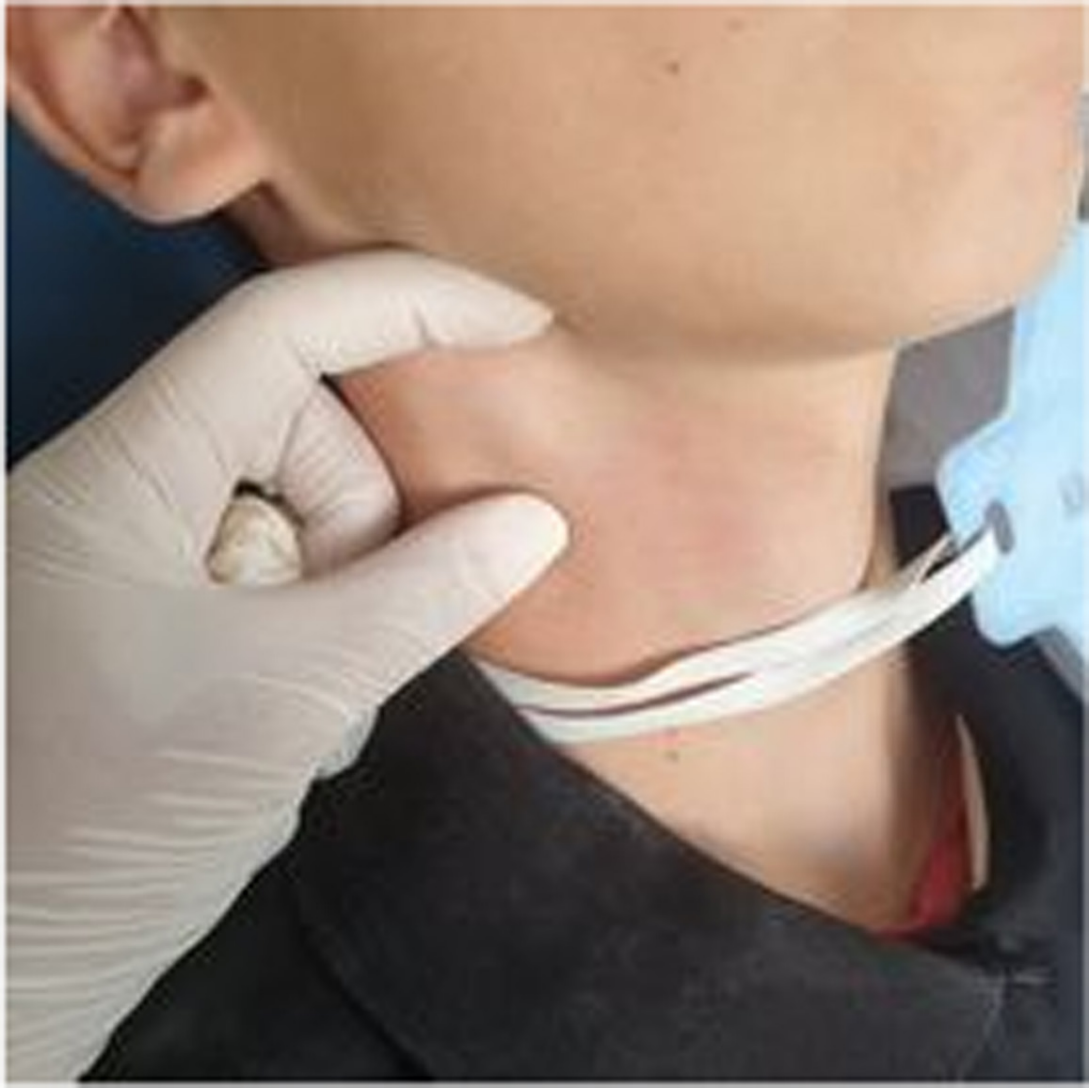
A right submandibular lymphadenopathy

**FIGURE 2:**
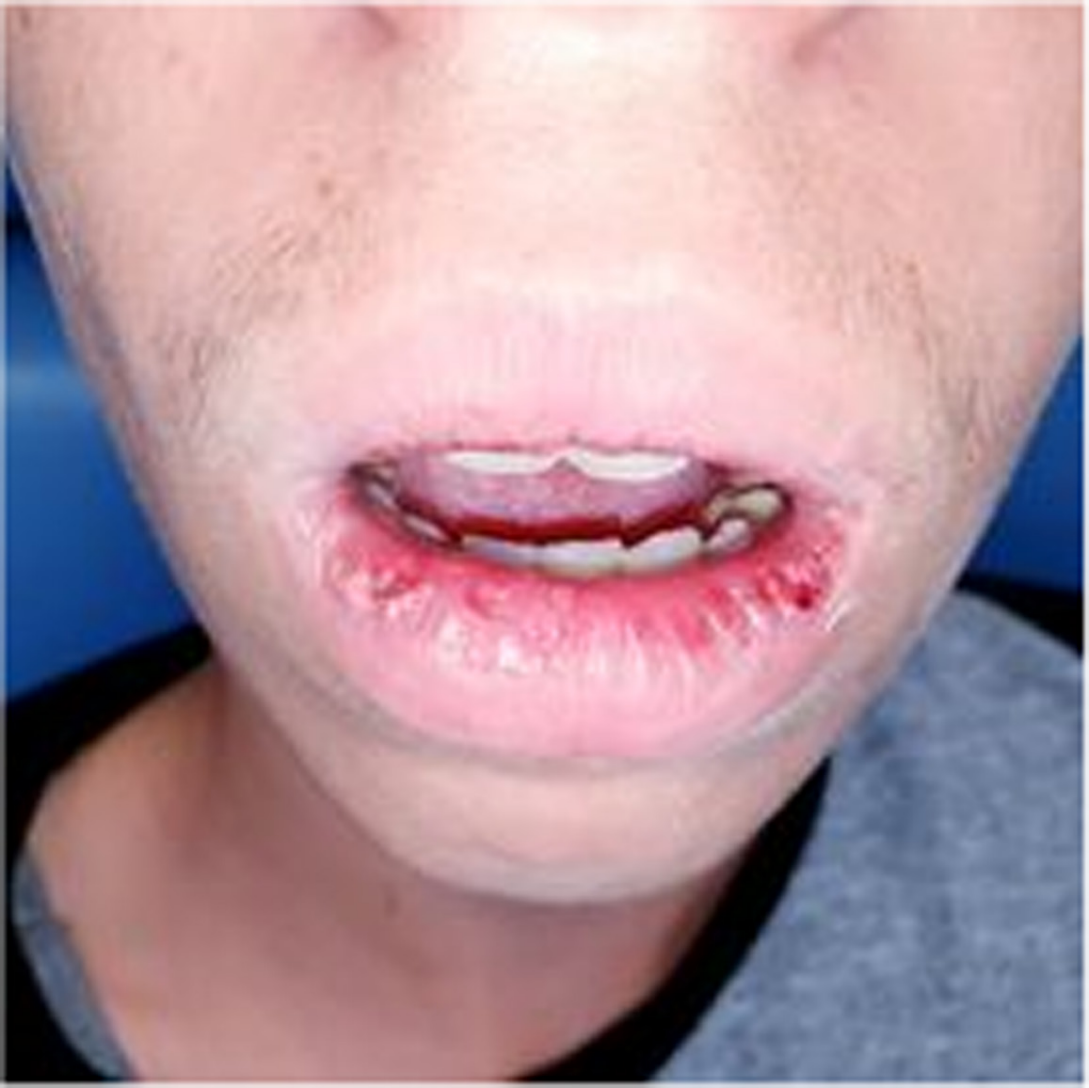
Erythema associated with ulcerations post blister in the lower lip

**FIGURE 3:**
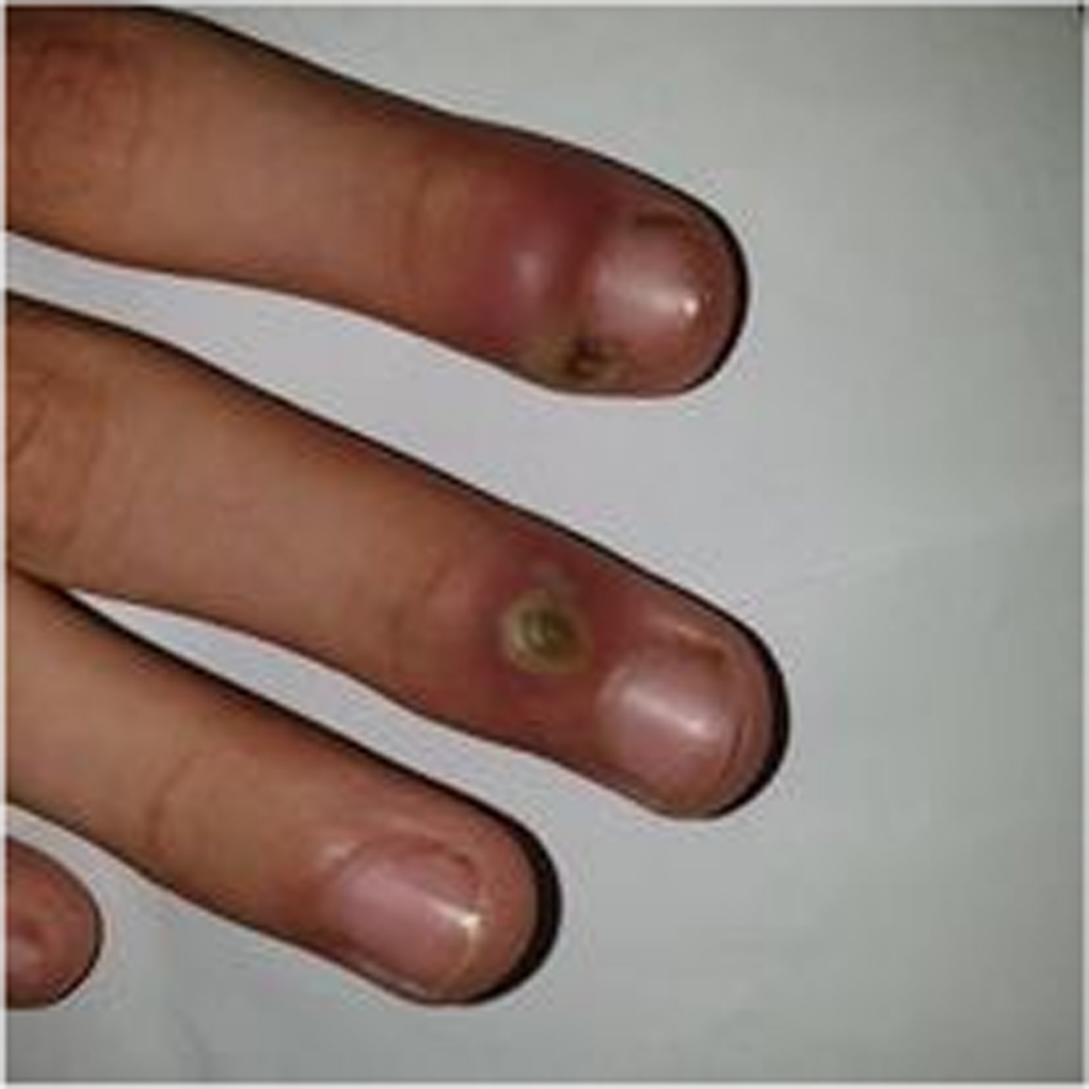
Herpetic withlow

**FIGURE 4:**
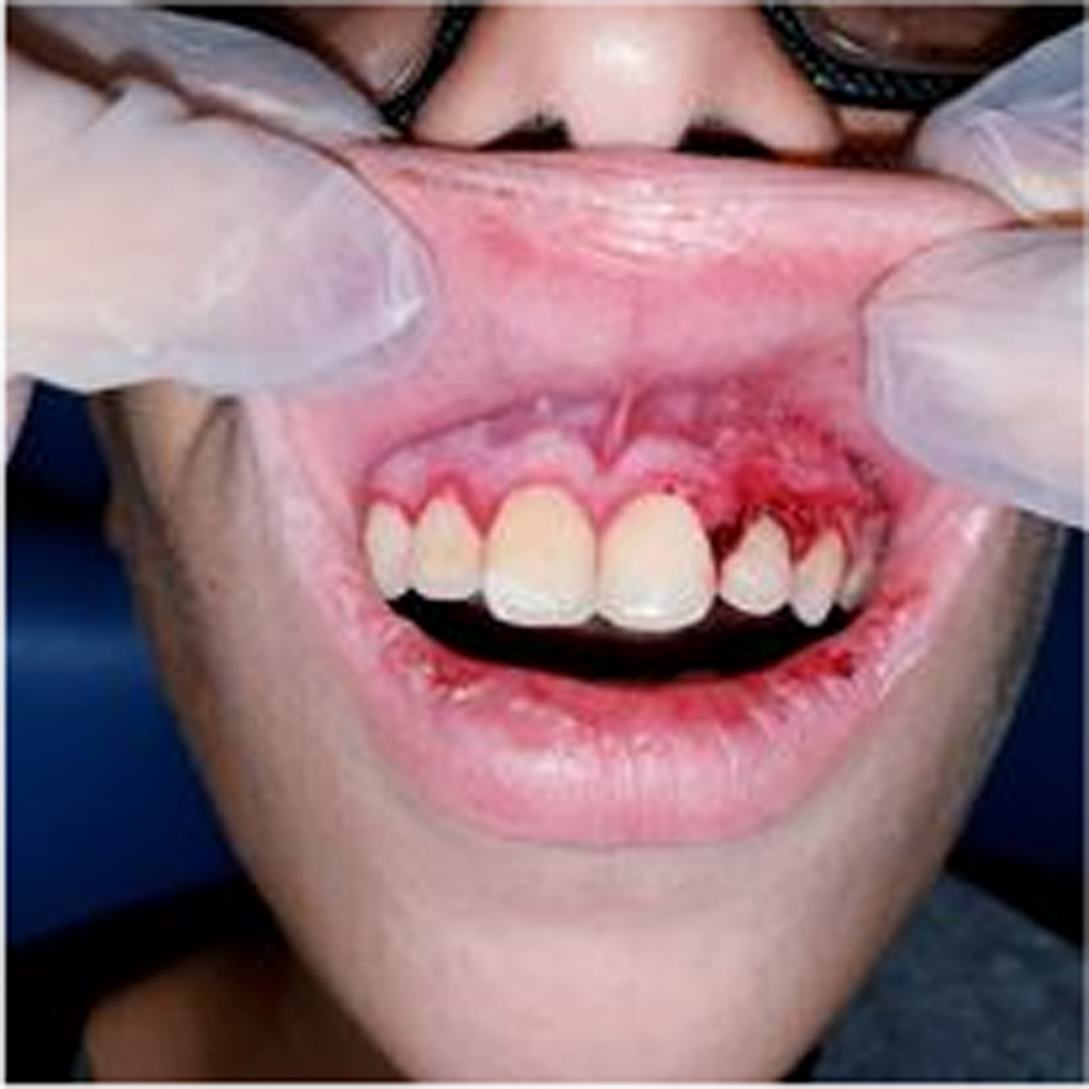
Marginal hemorrhagic gingivitis

**FIGURE 5:**
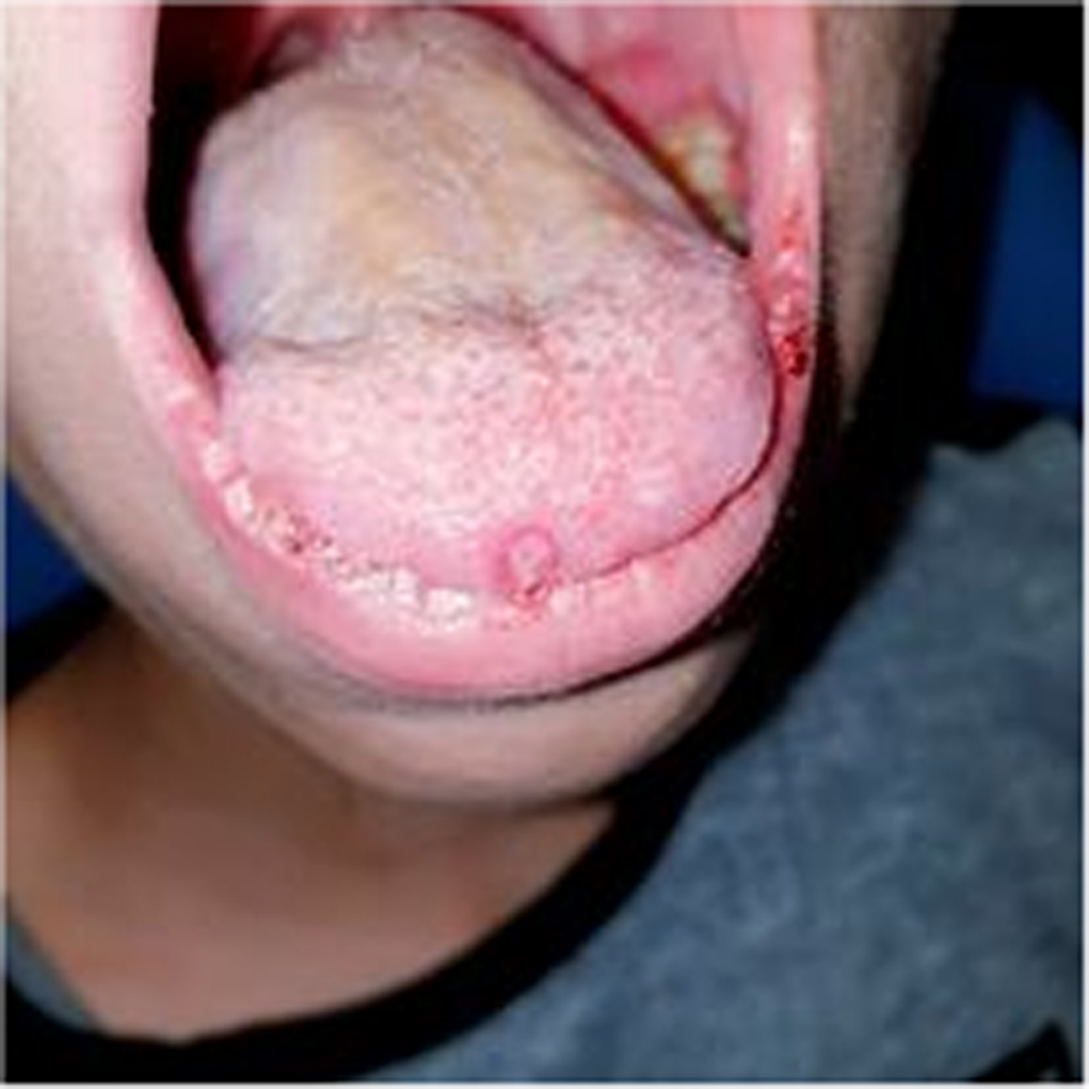
Ulcerations on the lateral edges of the tongue

A 10‐year‐old patient with no medical history consulted the department of oral medicine oral surgery at the University Dental Clinic of Monastir with the main chief complaint of food difficulty due to oral lesions developing for ten days. Medical history revealed dysphagia and asthenia associated with the apparition of the lesions. No medical or dental treatment had been taken to resolve the symptomatology. Extraoral examination revealed right submandibular lymphadenopathy which was measured 3 cm, mobile, painful on palpation, and covered with normal skin (Figure 1). Also, erythema associated with ulcerations post blister in the lower lip was noticed (Figure 2). There were small blisters at the extremity of the fingers. These remote lesions are called herpetic withlow that is caused probably by autoinoculation (Figure 3). The intraoral examination revealed an acute marginal hemorrhagic gingivitis (Figure 4), painful erosions in the maxillary gingiva, ulcerations on the lateral edges of the tongue (Figure 5), and post‐vesicular erosions in the palate. These clinical aspects are key features for a clinical diagnosis of acute primary herpetic infection.

**FIGURE 6:**
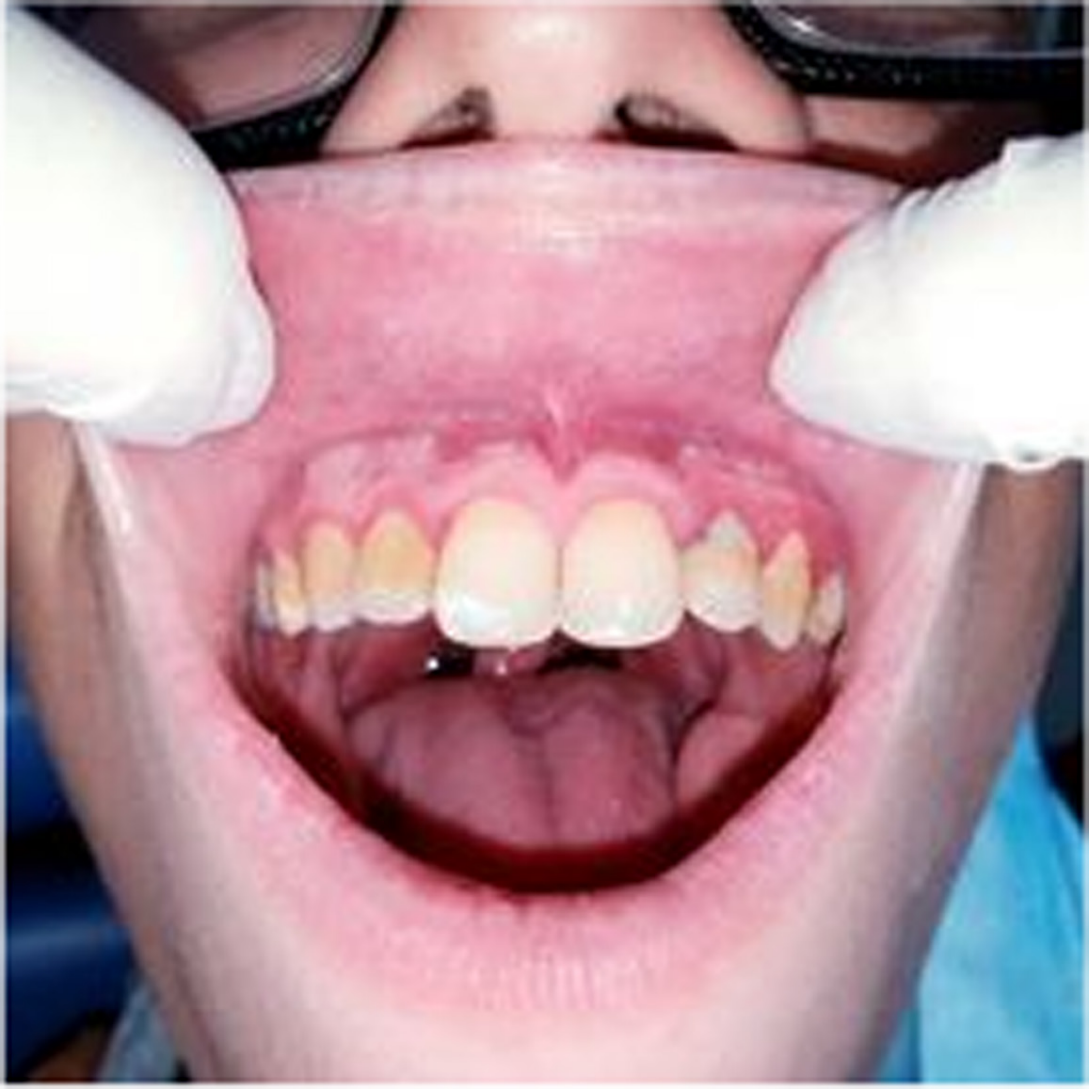
Regression of lesions

The main clinical differential diagnoses that were evocated in front of these lesions were childhood viral infections and military mouth ulcerations. We had prescribed an antibiotic, a Level 1 analgesic, anesthetic oral gel, and antiseptics in mouthwash. The patient responded well to the treatment showing regression of the symptomatology and disappearance of lesions after ten days (Figure 6).

## 3 DISCUSSION

Herpes simplex viruses (HSV) are DNA viruses belonging to the α‐herpesvirinae family. The human species is the only reservoir; the transmission is then only interhuman. There are 2 main types: HSV‐1 and HSV‐2, having a large degree of homology but differentiated by structural and epidemiological criteria. HSV‐1 is considered responsible for lesions above the waist, especially in those observed in the oral cavity and on the face. HSV‐2 is rarely associated with these regions and is most often responsible for infections of the genital tract.^4^ The prevalence of HSV‐1 infection increases with age. It is estimated about 70–80% in adulthood.^5^ Often the primary herpetic infection is not detected or incorrectly attributed by the parents to teething. However, in a small percentage of cases, the initial oral HSV‐1 infection is strongly symptomatic.^6^ Primary infection usually occurs in childhood. Eating difficulties and dysphagia expose to dehydration, which is considered the main complication. Besides, the patient may present other symptoms like fever, hypersialorrhea, and lymphadenopathy that last between 2 and 10 days. Pharyngitis, myalgia, asthenia, and irritability are also prodromal symptoms. Infected persons will often experience a pain, tingling, itching, or burning sensation around their mouth before the onset of the disease.^7^

The initial elementary lesions are vesicles, rarely seen in the mouth. These vesicles correspond to a small intraepithelial lift (<0.5 cm) caused by the death of infected keratinocytes.^8^ On the mucous membranes, the vesicles are fragile, and they break down quickly to become ulcerations with an erythematous border and are covered with yellow‐gray membranes, which can sit on all oral mucous membranes but mainly on the inner face of the cheeks, the tongue (dorsal side and ventral), the mucosal side of the lips and the gums. It is also accompanied or preceded by acute, generalized, hemorrhagic gingivitis. Another manifestation of herpetic primary stomatitis is an acute inflammation of the gingival margin and attached gingiva which is not accompanied by vesicular lesions. Inflammatory macroglossia is sometimes observed simultaneously.^9^ Herpetic geometric glossitis is described as an uncommon clinical manifestation of a primary HSV‐1 infection usually in immunocompromised patients. It is characterized by painful fissures on the dorsum of the tongue, with a branched pattern, with particular reference to the progression and complications of primary herpetic gingivostomatitis.^9^

Vesicles can be observed on the vermilion border and skin side of cheeks and chin. In the open air, the vesicles turn into scabs. The remote lesions are due to manual self‐inoculation. This is the herpetic whitlow caused by digital suction or the ocular herpes infection that occurs when the child rubs his eyes. Eye damage is a medical emergency. These lesions generally heal within 5–7 days while severe ones can require over 2–3 weeks. These symptoms can recur periodically, and the frequency varies from person to person.^10^

It has been described that PHGS is the key clinical feature of the primary HSV infection occurring in children, whereas pharyngotonsillitis, or mononucleosis‐like disease, is the primary HSV infection in adults.^11^ Gingivostomatitis in its typical form is evident, as the clinic is generally sufficient to confirm the diagnosis.^12^ However, evidence of the virus (culture, antigen) is necessary in certain situations such as atypical or complicated forms; newborns, immunocompromised patients or in case of meningoencephalitis (PCR value).^13^

Face a classic form of primary herpetic infection, we can evoke either childhood viral diseases such as chickenpox, herpangina foot–hand–mouth syndrome, military aphtosis, erythema multiforme, streptococcal pharyngitis, Behçet syndrome, Pemphigus vulgaris, acute necrotizing ulcerative gingivitis, candidiasis of the mouth or Steven–Johnson syndrome.^14^ On the contrary, childhood viral infections do not cause generalized acute gingivitis, and mouth vesicles are generally isolated. In addition, the good general condition, the absence of lymphadenopathies, and gingival ulcers make it possible to exclude the diagnosis of herpetic viral infection.^15^

In immunocompetent children, the primary herpetic infection evolves spontaneously to heal in 7–10 days without leaving any scars. Conversely, there are severe forms requiring hospitalization in newborns and immunocompromised children or undernourished.^8^

So, primary HSV‐1 infection in oral and perioral sites usually manifests as gingivostomatitis, whereas reactivation of the virus in the trigeminal sensory ganglion gives rise to mild cutaneous and mucocutaneous disease, often termed recurrent herpes labialis.^16^ Recurrent HSV‐1 infection in the mouth is less common than herpes labialis and unusual in otherwise healthy persons.

Treatment modalities of acute herpetic gingivostomatitis include supportive treatment with nutritional supplements and maintenance of fluids and electrolytes; antiviral medication and palliative therapy.

Systemic antiviral remedy has been extensively accepted as effective in reducing the symptoms of herpetic gingivostomatitis. The administration of antiviral medications (Acyclovir, Valacyclovir, Famciclovir) in the first 3–4 days after disease onset can effectively reduce the duration of the major symptoms like fever, oral ulcers, and food intake difficulty in children with PHGS [119]. Nevertheless, the optimal timing and cure of the treatment are uncertain.^17^ In fact, antiviral drugs can be used as a cream or as oral suspension administrated in a rinse. Acyclovir is considered as the gold standard because it is the most potent inhibitor of viral DNA polymerase.^18^

Sometimes, antiviral therapy is used to prevent or to reduce the frequency or the severity of the recurrence of herpetic infection in immunocompromised cases, but the optimal timing and duration of treatment are uncertain and can vary in different situations.^19^ Analgesics, anesthetics, or coating agents including lidocaine, diphenhydramine, milk of magnesia, Maalox, or Kaopectate can be used to relieve symptoms.^20^ Some authors advocate the use of a mouthwash consisting of diphenhydramine and magnesium hydroxide to accelerate the healing of the lesions.^21^ Some studies had shown that the low‐level laser therapy can be a good alternative as it reduces the severity of the symptoms and accelerates the healing.^22^

We should recommend children affected with primary herpetic gingivostomatitis to avoid sharing toys, pacifiers, and utensils used at mealtime unless they have been washed with water and soap and direct contacts with an immunocompromised person, elderly people, and pregnant women (2).

Besides, affected children should cut their nails to reduce the intensity of scratching and cover the cutaneous lesions with a hermetic bandage at night when possible.

Parents should be advised not to use products that contain benzocaine in infants or young children with primary herpetic gingivostomatitis, due to reports of benzocaine gel‐related cases of methemoglobinemia.

## 4 CONCLUSION

Clinically, the onset of PHGS is often very painful and debilitating. It may cause stomatitis lesions all over the oral cavity with the presentation of gingivitis (as gum swelling/bleeding), which are key features for a clinical diagnosis of PHGS. Meticulously identifying these specific oral manifestations can help make an adequate diagnosis earlier and subsequently reduce unnecessary prescription of antibiotics.

Although, it is a self‐limiting disease, an antiviral drug could be administrated ideally while the first 3 days to shorten the duration and severity of the lesions and to reduce the frequency of the recurrences.

## AUTHORS CONTRIBUTIONS

Khalifa Chaima has written the manuscript. Slim Afef has treated the patient and participated in the design as well as the revision of the manuscript. The other authors discussed the results by revising critically for important intellectual content and have given final approval of the version to be published. All authors approved the final draft of the manuscript.

## CONFLICT OF INTEREST

The authors declare that they have no conflicts of interest or sources of funding for this particular study.

## ETHICAL APPROVAL

Our institution does not require ethical approval for reporting individual cases or case series.

## CONSENT

Written informed consent was obtained from the patient for his anonymized information to be published in this article.

## Data Availability

The datasets generated during the current study are not publicly available but are available from the corresponding author on reasonable request.
